# On the Practical Study of Botany, by Means of Chemical Analysis:—Chiefly Abridged from the Original Papers of Dr. S. F. Hermbstaedt, Professor of Chemistry and Pharmacy, at Berlin

**Published:** 1799-03

**Authors:** 


### *On the practical Study of Botany, by means of Chemical Analyſis:—Chiefly abridged from the original Papers of Dr.* S. F. HERMBSTAEDT, *Profeſſor of Chemiſtry and Pharmacy, at Berlin.*

WHEN we contemplate the wonderful relation between cauſes and effects, every where ſubſiſting in the works of nature; when we compare the infinitely diverſified ſtructure of organized bodies with that of inanimate matter, our aſtoniſhment increaſes in proportion to the extent of our reſearches, and we are involuntarily led to diſcover a certain order and regularity, in ſpace and time, prevailing throughout the whole ſyſtem.

The moſt ſuperficial obſerver is forcibly ſtruck with the admirable ſucceſſion of plants, uniformly correſponding in their appearance, riſe, growth, &c. with the different ſeaſons; and although this *ſpontaneous* periodical effort of nature appears to be excluſively devoted to *vegetable* ſubſtances, it may ſometimes be traced, in a faint degree, even to the boundaries of the animal kingdom. Indeed, the line of demarcation between animal and vegetable life is but ſlightly marked, ſo that it remains to this day a matter of doubt, whether ſome of the marine plants, as well as the moving plant (*Hedyſarum motitans*, LINN.), and ſeveral ſpecies of *Tremellæ*, are not to be conſidered as ſubjects of the animal, rather than of the vegetable kingdom.

Although the *animal* world be likewiſe governed by fixed laws, relative to the ſize and form of bodies, yet it may be aſſerted that nature here has reſigned reſigned part of her authority to the will and inclinations of the creature:—neither the ſucceſſive periods of propagation, nor the limits of duration, are diſcriminated with the ſame accuracy as is perceptible, in every ſeaſon, in the vegetable world.

The *mineral* kingdom, with all the riches, beauties, and ſportive productions it contains, is the leaſt perfect of the three; as the principal circumſtance which characterizes this part of nature's wonders, conſiſts in the peculiar ſtructure and exact form of cryſtalizations, the ſize of which is as indefinite, as their origin is accidental, ſo that the formation of cryſtals apparently depends more upon *chance*, or a ſort of whimſical combination of circumſtances, than upon the premeditated deſigns of nature.

It is well-known to every chemiſt, what an endleſs variety of cryſtalizations may be produced artificially, by combining different ſalts and acids with each other, and cauſing them again to cryſtalize. We can, in ſome degree, imitate this proceſs in vegetable ſubſtances, and thus likewiſe artificially multiply the variety of plants and trees; but the genera and ſpecies are not ſubject to laws, either arbitrary or accidental, as they muſt neceſſarily remain in the ſtate appointed them by nature, unleſs diſpoſed otherwiſe by the hands of men.

From theſe deſultory reflexions we may rationally infer, that the inveſtigation of plants and trees, with a view to obtain a more perfect knowledge of their regular organic ſtructure, juſtly deſerves the attention of every inquiſitive mind. Curioſity alone is, with ſome perſons, a ſufficient inducement to apply themſelves to the ſtudy of botany, the moſt charming part of Natural Hiſtory. ROUSSEAU expreſſes himſelf, on this ſubject, with his uſual happy diſcernment, in the following words: "I am perſuaded" ſays he, "that at all times the ſtudy of nature checks the ardour for frivolous amuſements, prevents or aſſuages the tumult of the paſſions, and provides the mind with a moſt ſalutary nouriſhment, by filling it with an object worthy of its contemplation."

But the principal advantages to be derived from a *proper* ſtudy of this delightful ſcience, are not merely thoſe of affording pleaſure and innocent occupation to the mind, as eloquently urged by the philoſopher of Geneva: they are of a ſtill more extenſive and *practical* nature, and claim the attention of all claſſes, and even of every member of the community.

It certainly muſt afford us a degree of rational entertainment, when we are enabled to diſtinguiſh the variegated productions of vegetable nature, as to their form, ſtructure, ſize, native ſoil, and other circumſtances connected with the periods of their birth and diſſolution. Thus we learn to arrange them into claſſes, orders, orders, genera, and ſpecies; to point out, in the ſyſtem, the exact place of every new plant which may yet be diſcovered, and hereby ſave ourſelves much trouble and confuſion. This however may be termed merely ſcholaſtic, or a mechanical kind of knowledge, ſo long as we remain ignorant of the various conſtituent parts of the plants themſelves, and the many important uſes and purpoſes, to which they may be daily applied in rural, domeſtic, and medical economy, as well as in ſome of the moſt valuable departments of the arts and manufactures.

The late diſcoveries in chemiſtry have ſufficiently ſhewn, that the component parts of vegetable bodies are of a very compound nature, and that conſequently their *chemical analyſis* requires a greater degree of accuracy and perſeverance, than has hitherto been beſtowed on this important branch of phyſics. We ſhall find upon a cloſer inquiry into the nature of vegetable productions, that we muſt recogniſe or admit ſuch a number of *diſtinct elementary principles* as we can diſcover and exhibit to the ſenſes by well-conducted experiments. For, if certain parts which enter into the compoſition of plants, can be ſeparated from them in a great variety of inſtances; if they are manifeſt to the obſerver, not only as diſtinct ſubſtances, but alſo as chemically related to other bodies—we have in that caſe, doubtleſs, a right to conſider them as diſtinct elementary parts. Such a diſtinction of the various uſeful ingredients with which nature has richly provided her vegetable children, is of eſſential conſequence; as it will lead us in many caſes to determine with accuracy their reſpective uſe and value. But, as their utility is not confined to *medicinal* purpoſes *only*, it will be neceſſary to take a more comprehenſive view of the ſubject under conſideration, and to arrange our materials more ſyſtematically, than our predeceſſors were inclined or able to do, from their acknowledged deficiency in chemical knowledge.

The *bare* arrangement of vegetable productions, together with the moſt perfect acquaintance with the names and *botanical* characters of plants, is an acquiſition of only relative value, conſidering the ſtudy of botany in a *practical* ſenſe; that is, ſo far as it is more immediately connected with the uſeful purpoſes of life. In this reſpect, an immenſe treaſure at once opens to our view. And it may be confidently aſſerted, that botany is of greater practical utility than any other ſcience; for, if the knowledge of vegetable ſubſtances be eſtabliſhed upon a *chemical baſis*, it may be rendered ſubſervient to the moſt important purpoſes; a truth which will be amply demonſtrated by the following reaſons and illuſtrations.—No ſacrifice of time and labour will be found adequate to the important advantages, which may be derived from an accurate analyſis of plants, and their extenſive application to health

œconomy

œconomy, and the arts of dying and painting, weaving, brewing, diſtilling, tanning, ſoap-making, &c. &c: indeed, theſe advantages are not limited to any particular claſs or rank of ſociety; they are as manifold and beneficial, as the productions of the vegetable kingdom are in themſelves wonderful and innumerable. The infinite variety of experiments that can be inſtituted with vegetable ſubſtances, as they are highly gratifying to the laudable curioſity of minds of an inquiſitive and ſcientific turn, ſo are they equally intereſting, for profitable purpoſes, to the huſbandman, the artiſt, and the manufacturer; while the young and the gay alſo find their account in them, as they tend in a great meaſure to controul the rage for diſſipation, and gradually to habituate the admirers of nature to more ſerious and uſeful purſuits. To prove and eſtabliſh what is here advanced, we propoſe to exhibit the various *primary* and *ſecondary* conſtituents of vegetable bodies, together with the *acids* which may be obtained from them, in *three* ſeparate *tables*. And after having indicated theſe particulars, we ſhall endeavour to furniſh the reader with a *complete* liſt of chemical teſts, or re-agents, by means of which the preſence or abſence of certain ingredients or properties, inherent in the various parts or productions of plants and trees, may be moſt effectually diſcovered, and their proportionate quantity aſcertained with preciſion.

### TABLE FIRST.

*Primary Elements of Vegetables which can be ſeparately exhibited as Objects of Senſe, without undergoing any Change of their native or inherent Qualities.*

1. THE ſubſtance of *Gum*, (as being different from mucilage and reſin,) for inſtance, the pure *Gum Senegal;* which is obtained from the *Mimoſa Catechu Linn.*, and which by the old chemiſts was improperly called *Terra Japonica.*—The pure ſubſtance of this gum agrees with that of every plant, if obtained in a pure ſtate; it is perfectly ſoluble in water; the inſpiſſated ſolution of it may be drawn in threads, and it is, upon the whole, extremely glutinous.

2. *Mucilage.* Nature furniſhes this ſubſtance, in a pure ſtate, in the *Aſtragalus Tragacanthus, Linn.* (or more properly the Aſtragalus *Creticus,*) which is to this day erroneouſly called *Gum Tragacanth,* although it affords by no means a perfectly tranſparent ſolution in the pureſt water; on the contrary, it remains always opaque, does not feel glutinous but ſlippery between the fingers, and cannot be drawn in threads. The farinaceous or mealy part of vegetables, in its *pure* ſtate, is likewiſe a mucilaginous ſubſtance; it ſhould be obſerved, however, that true meal always implies and contains ſtarch, *amylum,* which forms the principal part of the farina.

3. True

3. True *Reſin*, the characteriſtic of which is, that it makes a perfect ſolution in ſpirit of wine, or vitriolic ether. The *pure* ſubſtance of reſin is of an homogeneous nature in all inſtances, ſo that the foreign ingredients occaſionally found in different reſins, conſtitute all the difference obſervable in them.

4. *Soap*, or ſaponaceous matter, a neutral ſubſtance between gum and reſin.—This name ſhould be excluſively appropriated to a certain principle in plants, which is ſoluble in the pureſt water, as well as in the moſt rectified ſpirit of wine, but is not acted upon by vitriolic ether. Thus, the conſtituent parts extracted from ſaffron almoſt entirely conſiſt of ſaponaceous matter, which is a very copious ingredient in the compoſition of vegetable bodies; but, on account of its eaſy ſolubility, both in water and ſpirits, it has hitherto been as often confounded with reſins as with gums. It is one of the moſt general conſtituents of vegetables, and hence promiſes conſiderable advantages to the manufacturer of this article, as it is no leſs ſubſervient to the purpoſes of domeſtic economy.

5. *Sugar*, or the ſaccharine principle. It exiſts in all vegetable ſubſtances of a ſweet taſte, but is ſo intimately blended with gummy, mucilaginous and ſaponaceous matters, that it cannot be ſeparated from them without ſome difficulty.

6. *Albumen*, firſt ſo named by Fourcroy, on account of its exact reſemblance to the white of eggs. It is found principally in creſſes, ſcurvy-graſs, hemlock, and moſt abundantly in the antiſcorbutic and narcotic plants, where it geuerally reſides in the leaves. Its preſence may be eaſily diſcovered, by mixing the freſhly-expreſſed juice of theſe plants with ſpirit of wine, or by macerating them in hot water, nearly to the boiling point: in both theſe caſes the albumen will coagulate and ſeparate itſelf from the other fluids in the form of cheeſy matter.

7. *Oil*, or the baſis of every etherial and diſtilled oil. It differs from the following ſubſtance (No. 8.) merely in the proportion of its conſtituent parts. According to the opinion of Hermbſtaedt, the baſis of vegetable *oil* is carbon and hydrogen only; whereas that of *fat* conſiſts of carbon, hydrogen, and oxygen. Vegetable fat may be converted into oil by means of dry diſtillation; but, whether the baſis of oil does not alſo contain the *odoriferous* principle in vegetables, or whether this be combined with ſome other element (ſuch as that of *Boerhaave's Spiritus rector*) is a point not yet determined, and remains to be illuſtrated by future experiments.

8. *Fat* is the baſis of every vegetable oil. The difference between oils and fats, in general, conſiſts merely in the foreign ingredients with which the the mater of fat is combined. The oil of almonds, and the butter of cocoa, in the opinion of our author, are both of them ſubſtances purely fatty, and they differ only as to the degrees of conſiſtency.

9. *Camphor* has hitherto been conſidered as a peculiar ſubſtance, different from all other vegetable matter, though in its properties it moſtly reſembles the oleaginous bodies. Some vegetables contain, beſides the matter of oil, a conſiderable portion of camphor in their conſtituent parts; the preſence of which may be frequently diſcovered by an odour and pungent taſte, accompanied with a ſenſation of coolneſs on the tongue; this is found in the peppermint, ſage, cardamom, and other ſubſtances.

10. *Wax* appears to be different from the matter of fat, in the ſame degree as oil is from that of camphor. The preſence of wax is diſcoverable in a much greater number of vegetables than has hitherto been ſuppoſed; it may be obtained from the leaves of moſt plants and trees, as is manifeſt from their ſhining cover or varniſh, which generally conſiſts of waxy matter. It is alſo met with as a conſtituent part of ſeveral ſorts of reſins, and may be ſeparated from gummy, mucilaginous, and ſaccharine matters, by means of mere water; from ſaponaceous ſubſtances, by water or ſpirit of wine; and from reſinous bodies, by means of vitriolic ether.

11. *Elaſtic Gum.* Although this name is by no means the moſt eligible, it muſt remain here until we can ſubſtitute a better. This valuable ſubſtance, which undoubtedly forms a conſtituent part of many vegetables, appears to be moſt intimately combined with the matters of reſin, albumen, and fat; hence it is extremely difficult to diſengage it from them by art. Its proper ſolvent is a pure vitriolic ether; but a ſtill better one has been diſcovered lately, which is rectified *petroleum*, or rock-oil; in this it is perfectly ſoluble, and may again be ſeparated from it, by ſimple diſtillation with water. In the analyſis of vegetable bodies, the elaſtic gum has till lately been almoſt entirely overlooked.

(To be continued in our next Number.)

MEDICAL

